# Identification and characterisation of constitutional chromosome abnormalities using arrays of bacterial artificial chromosomes

**DOI:** 10.1038/sj.bjc.6601588

**Published:** 2004-02-17

**Authors:** J K Cowell, Y D Wang, K Head, J Conroy, D McQuaid, N J Nowak

**Affiliations:** 1 Department of Cancer Genetics, Roswell Park Cancer Institute, Elm and Carlton Streets, Buffalo, NY 14263, USA

**Keywords:** array-CGH, chromosome deletion, cancer syndromes, BACs

## Abstract

Constitutional chromosome deletions and duplications frequently predispose to the development of a wide variety of cancers. We have developed a microarray of 6000 bacterial artificial chromosomes for array-based comparative genomic hybridisation, which provides an average resolution of 750 kb across the human genome. Using these arrays, subtle gains and losses of chromosome regions can be detected in constitutional cells, following a single overnight hybridisation. In this report, we demonstrate the efficiency of this procedure in identifying constitutional deletions and duplications associated with predisposition to retinoblastoma, Wilms tumour and Beckwith–Wiedemann syndrome.

Predisposition to a variety of cancer predisposition syndromes occurs as a result of the inheritance of gains and losses of chromosomes and chromosome regions. Currently, Giemsa-banding analysis of constitutional chromosomes is the most usual way of identifying these chromosome abnormalities. Typically, these cytogenetic approaches can readily identify whole chromosome changes as well as intrachromosomal deletions, with a maximum resolution of approximately 10 megabase (Mbp). The limitation of these analyses is that they cannot always reliably detect small chromosome deletions or amplifications. If the regions of the chromosome involved are known, for example, based on the clinical features of the syndrome, it is possible (Kempski and Cowell, 1993; Cowell *et al*, 1994) to analyse the chromosomes using fluorescence *in situ* hybridisation (FISH). Duplications of small chromosome regions are harder to identify by FISH, especially if the duplication is small and tandem. The limitation of this FISH approach is that it is usually performed on a locus-by-locus basis, which requires some clinical indication of the part of the genome that is involved. Identifying the specific chromosome abnormality in these patients can be important for the clinical management of the patient, since the larger the deletions the more severe the associated clinical phenotypes.

Despite the limitations of karyotype analysis, it has been the cornerstone for providing the diagnosis of human chromosome-related syndromes. A method that unequivocally identifies the presence of the chromosome changes in a nonbias analysis and also defines the exact region involved directly from DNA samples in a relatively short time, would address most of the shortcomings of cytogenetics-based approaches. Over the past several years, we have been developing a hybridisation approach that allows an analysis of chromosome deletions and amplifications, without the need to study metaphase chromosomes. This approach is referred to as array comparative genome hybridisation (CGHa), and consists of a series of mapped BACs arrayed on a glass slide to which DNA from test and control samples are competitively hybridised (Hodgson *et al*, 2001; Snidjers *et al*, 2001; Cowell and Nowak, 2003).

In previous reports, CGHa has been used in highly focused studies using limited sets of BACs, but only from well-defined regions of the genome. Thus, Veltman *et al* (2002) used CGHa arrays which comprised <100 BACs from the subtelomeric regions of the human chromosomes to identify subterminal deletions. Similarly, Veltman *et al* (2003b) used approximately 100 BACs from chromosome 18 to specifically investigate the deletions associated with congenital aural atresia, and approximately 100 BACs from chromosome 22 were used to identify deletions of the NF2 gene in NF2 patients (Bruder *et al*, 2001). In these cases, it was necessary to know where in the genome to look and, if there has been any other genetic change in the genome, these would be missed. Larger arrays have been used in the analysis of a number of different cancers, but again the level of resolution has been in the range of 1–1.5 Mbp or less. Thus, Wessendorf *et al* (2003) used an array of approximately 500 BACs to study B-cell non-Hodgkin lymphoma, but these BACs only sampled regions of the genome which had previously been shown to be involved in this malignancy. Arrays containing approximately 2000 BACs were used to study renal cell cancer (Wilhelm *et al*, 2002), bladder cancer (Veltman *et al*, 2003a) and a number of cell lines (Fiegler *et al*, 2003), and could identify large genetic changes. The resolution of the BAC arrays, however, is an important consideration, since more dense arrays will detect smaller deletions and amplifications, which, in turn, provide the best opportunity to define the driver gene for the abnormality.

In this report, we describe an array of 6000 BACs, which provide an average inter-BAC interval across the genome of 500 kb. In a single hybridisation, using as little as 50–100 ng, numerical chromosome abnormalities can be identified over the whole genome at a far greater resolution than previous reports (Cowell *et al*, 2003, 2004a, 2004b; Cowell and Nowak, 2004). We have used these CGH arrays to investigate their ability to define constitutional chromosome abnormalities associated with a number of different syndromes carrying deletions and duplications of varying size. In all cases, the specific abnormalities could be detected, which supports the idea that CGHa for this application could replace conventional karyotype analysis for most of the cancer predisposition syndromes that result from structural chromosome abnormalities.

## MATERIALS AND METHODS

DNA samples were prepared from lymphoblastoid cell lines previously derived from peripheral blood leucocytes. These cell lines were cultured in RPMI medium supplemented with 10% foetal calf serum and 10 mM glutamine.

### BAC array generation

A genome-wide resource of ∼6000 FISH mapped, gene/marker content verified, and sequenced BAC clones (Cheung *et al*, 2001) from the RPCI-11 human BAC library are represented as immobilised DNA targets on glass slides for array-based CGH analysis). Each clone is spotted in duplicate at 280 *μ*m intervals (see http://genomics.roswellpark.or g for a complete list of clones). The average inter-BAC interval on the array is approximately 500 kb, although the regions flanking the centromeres of all of the chromosomes are relatively under-represented, because of the high density of repetitive elements.

### DNA preparation

**Figure 1:**
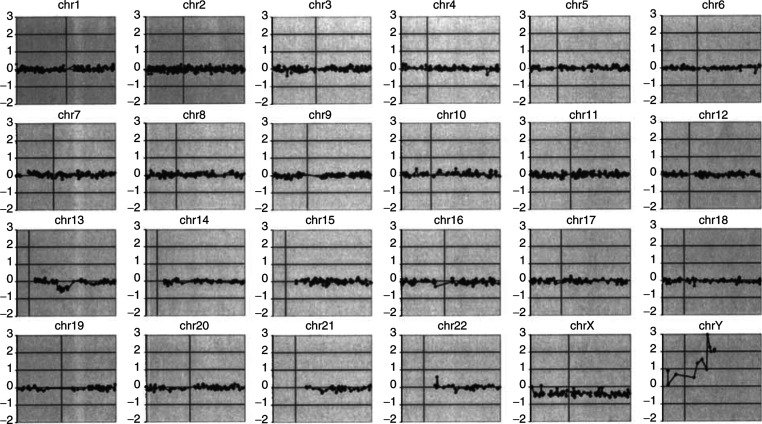
CGHa profile of constitutional DNA from patient GOS 115. The individual BACs for all chromosomes show a test/control ratio about a mean of 0 (no change, log scale), with the exception of the 13q14 region where the ratio is −0.5, indicating the presence of a heterozygous deletion (see text). The sex chromosome mismatch for this male patient was an XX control, which demonstrates a ratio or −0.5, which is expected in this experiment for a hemizygous deletion.

Genomic DNA was prepared from all samples using the FlexiGene DNA Isolation kit (Qiagen, Inc.). according to the manufacturer's instructions. Two control DNA pools are used for BAC CGH array analysis. The male control and female control pools each contain DNA from 15 cytogenetically normal individuals. For procedural quality control, all analyses are performed as sex-mismatch hybridisations. This allows determination of chromosome X and Y copy number as an internal reference standard (see Figure 1

).

### Labelling of DNA

A measure of 1 *μ*g of control and test genomic DNA was random primer labelled using a BioPrime DNA labelling kit (Invitrogen, Inc.) for 3 h at 37°C, with the appropriate Cyanine dye (Cy3 or Cy5). After ethanol precipitation, the probes are resuspended in H_2_O and combined. Unincorporated Cy dye is removed by passage over a Qiagen spin column. The labelled probes are dried and stored at −20°C until hybridisation.

### Hybridisation

Briefly, the arrays are preblocked with 110 *μ*l Ambion SlideHyb Buffer #3, 1 *μ*l of 20 *μ*g *μ*l^−1^ Human Cot-1 DNA solution at 50°C in a GeneTAC hybridisation station (Genomic Solutions, Inc.) for 30 min. Prior to hybridisation, the probe is resuspended in 110 *μ*l Ambion SlideHyb Buffer #3 containing 5 *μ*l of 20 *μ*g *μ*l^−1^ Cot-1 and 5 *μ*l of 100 *μ*g *μ*l^−1^ yeast tRNA, heated to 95°C for 5 min and placed on ice. The prehybridisation buffer is removed, the entire probe added to the hybridisation chamber, and hybridisation proceeds for 16 h at 65°C in the GeneTAC. After hybridisation, the slide is washed in decreasing concentrations of SSC and SDS, followed by one 0.1 × SSC wash, one 95% EtOH rinse and centrifugal drying for 3 min.

### Image analysis

The hybridised slides are scanned using an Affymetrix 428 Scanner to generate high-resolution (10 *μ*m) images for both Cy3 and Cy5 channels. Image analysis is performed on the raw image files using ImaGene (V4.1BioDiscovery). Each spot is defined by a circular region, the size of which is programmatically adjusted to match the size of the spot. A buffer region of 2–3 pixels around the spot is ignored and a region 2–3 pixels wide outside the buffer region is considered the local background for that spot. Each spot and its background region are segmented using a proprietary optimised segmentation algorithm, which excludes pixels that are not representative of the rest of the pixels in that region. The background corrected signal for each BAC is the mean signal (of all the pixels in the region) minus the mean local background. The output of the image analysis is in the form of two tab-delimited files, one for each channel, containing all of the fluorescence data.

### Data analysis

The output of the image analysis is processed by a program written in Perl and R, developed at RPCI. For each spot, the ratio is calculated from the background subtracted mean signal of the two channels. The ratios are then normalised on the log scale with a nonlinear normalisation algorithm. Basically, for all spots that are flagged as having met the qualitative spot criterion, the log_2_ background subtracted mean signal is plotted and a lowess function is applied. The normalised ratios are the computed ratios minus the expected values on the curve.

The results of the triplicate replicas are combined by taking the mean of the log_2_ ratios and the standard error is calculated. Any BAC that has less than two replicates flagged as having met the qualitative spot criterion is excluded. Mapping information is added to the resulting ratios and standard errors. The mapping data for each BAC are found by querying the human genome sequence at http://genome.ucsc.edu. The Nov 14 2002 build is currently being used to precisely position the BAC clones on the draft sequence. The output, a tab delimited file, is imported to Excel for graphing.

### Interpretation

The final ratio represents the relative amounts of DNA from the experimental sample and the reference control sample. Equal amounts of control and test DNA are labelled and the ratios are normalised to 1 (0 on the log scale), effectively normalising the array to the average modal number of the test sample. Knowledge of the sex of the test sample is used to perform a sex mismatch between the test sample and the control, providing an internal control for copy number. Typically, a degree of suppression is observed in these ratios. The X chromosome, therefore, can be used to estimate the amount of suppression when the test sample has a normal number of sex chromosomes, that is, XX or XY.

## RESULTS AND DISCUSSION

Using conventional cytogenetics retinoblastoma (Rb), patients with mental retardation and dismorphic features have been typically shown to carry deletions on the long arm of chromosome 13 involving the 13q14.3 region (Cowell *et al*, 1989a). These deletions eliminate one copy of the retinoblastoma predisposition gene (RB1). Small deletions, however, do not have the same range of diagnostic clinical features, and screening all Rb patients cytogenetically is cost prohibitive, since less than 10% will carry deletions (Cowell *et al*, 1989a). The esterase-D (ESD) gene was localised adjacent to RB1 (van Heyningen *et al*, 1975), such that deletions involving this region could be identified by measuring ESD activity in red blood cells from the patient. To identify these deletions, we developed a screening procedure which involved measuring the ESD activity in retinoblastoma patients (Cowell *et al*, 1986a). Since the ESD gene lies approximately 650 kb centromeric to RB1 (Young *et al*, 1988), this was the only chromosome-independent approach for the detection of deletions at that time. Esterase-D quantitation for the discovery of deletions was occasionally confounded by the possibility that the proximal breakpoint of the deletion separated the ESD and RB1 genes (Cowell *et al*, 1987; Mitchell and Cowell, 1988). Although ESD quantitation proved to be a very quick and effective screening tool (Cowell *et al*, 1986a), the low endogenous activity of the rarer ‘2’-allele meant that 2–2 homozygotes would often produce enzyme levels in these patients that were close to 50% of the normal controls (Cowell *et al*, 1986b). This was particularly problematic in groups such as the Japanese population (Horai and Matsunaga, 1984), where the incidence of the 2-allele is significantly greater (40%) than in Caucasian (10%) populations (Cowell *et al*, 1986a).

**Figure 2:**
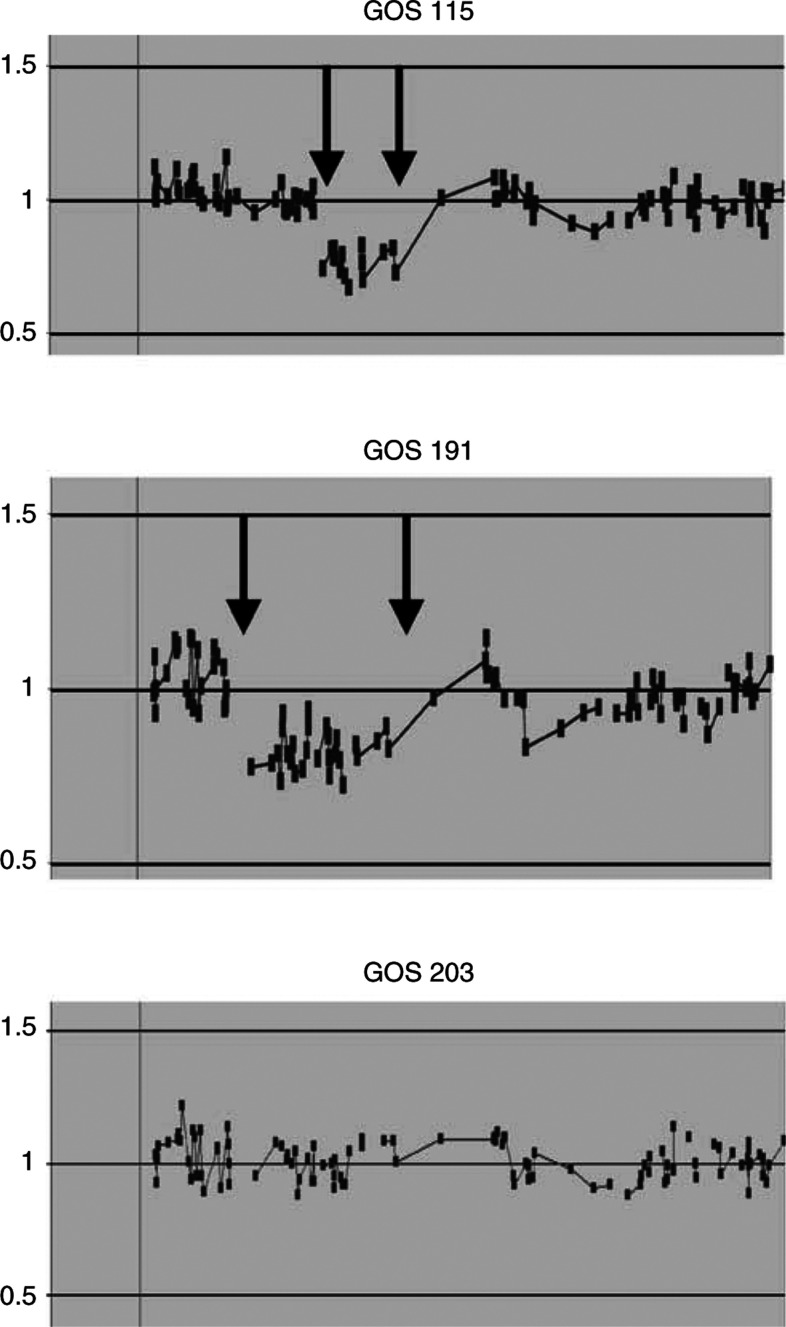
CGHa profile for patients with retinoblastoma. The deletions (arrows) in 13q14 for GOS 115 and 13q14–22 in GOS 191 can be clearly seen with a ratio of approximately –0.5. Patient GOS 203 shows a normal profile for chromosome 13 with all BACs, clustering around a mean of 1 (linear scale).

To assess the ability of the CGH BAC arrays to identify 13q14 deletions in Rb patients, we used DNA to perform the hybridisation derived from a series of lymphoblastoid cell lines (Cowell *et al*, 1989a) established from representative Rb patients with deletions of varying length. An example of a complete genomic profile that is produced from the 6000-clone BAC array is shown in Figure 1. All of the chromosomes show clustering of the hybridisation ratios about the mean of 0 (diploid on the log_2_ scale). The heterozygous deletion in two patients (GOS 115 and GOS 191) was readily identified as the only abnormality in the sample (Figure 2

). The deletion associated with patient GOS 191 was cytogenetically easily detectable (Cowell *et al*, 1989a) and this deletion was shown by CGHa to extend between BACs RP11-11k16 (32.04 Mbp) and RP11-37i864 (64.09 Mbp), which, based on the human genome sequence, represents a distance of 32.05 Mbp. The deletion associated with patient GOS 115, however (Figure 2), was more subtle (Cowell *et al*, 1989a), and was seen as a subband deletion surrounding the RB1 locus. Our CGHa analysis demonstrates that, in fact, this deletion spans the region of 13q14 between BACs RP11-20k19 (44.86 Mbp) and RP11-37i8 (64.09 Mbp), which constitutes maximally 20 Mbp. This analysis, therefore, also provides an approximate relationship between the DNA sequence and the appearance of deletions in metaphase chromosomes at the 850-band resolution. The RB1 gene is located at position 47.81–47.99 Mbp along the long arm of chromosome 13.

The CGHa data demonstrate some important empirical details for interpretation of the profiles. The prediction from the XX/XY mismatch is that, on the log scale, there should be a ratio of −1 for DNA from males and +1 for females. In fact, we consistently see that this ratio is closer to ±0.5. Although we do not know the full cause of this suppression of the hybridisation ratio, we presume that much of it is due to nonspecific hybridisation in the system, most likely due to repetitive sequences which cannot be competed out using *Cot*1 DNA (Cowell and Nowak, 2003). Despite this reduction in hybridisation ratio, however, it is clear that deletions and duplications (see below) show a change in ratio that is consistent with that seen for the X chromosome in the same experiment, which makes definition of the chromosome change relatively easy.

During our analysis of retinoblastoma patients using ESD screening, we identified a potential chromosome deletion in patient GOS 203, where the enzyme levels were 50% of that seen in normal controls (Cowell *et al*, 1986b). This patient was shown, using starch gel electrophoresis, to be homozygous for the ‘2’ allele, which we had already shown had an inherently lower activity than the 1-allele. Heterozygotes show reduced levels (Cowell *et al*, 1986b), but not large enough to suggest a 50% reduction in activity. Patient 203 showed mild dismorphic features and reduced cognitive ability. Chromosome analysis, however, appeared normal but did not, together with the ESD levels and clinical phenotype, rule out the presence of a submicroscopic deletion unequivocally. Clearly, although a rare case, genetic counseling in this family was inadequate, since we could not ignore the potential that she carried a deletion based on the enzyme assays. These deliberations were tempered by the lack of convincing cytogenetic data and the mild clinical phenotype. The CGHa profile for chromosome 13 from this patient is shown in Figure 2, and clearly shows diploid levels along the length of the chromosome and in particular for BAC RP11-174i10, which contains the RB1 gene. This result formally demonstrates that this patient does not carry a 13q14 deletion. Although it has been some time since genetic counselling was given to this patient, the ESD result clearly influenced this family in their choice not to have children at the time.

**Figure 3:**
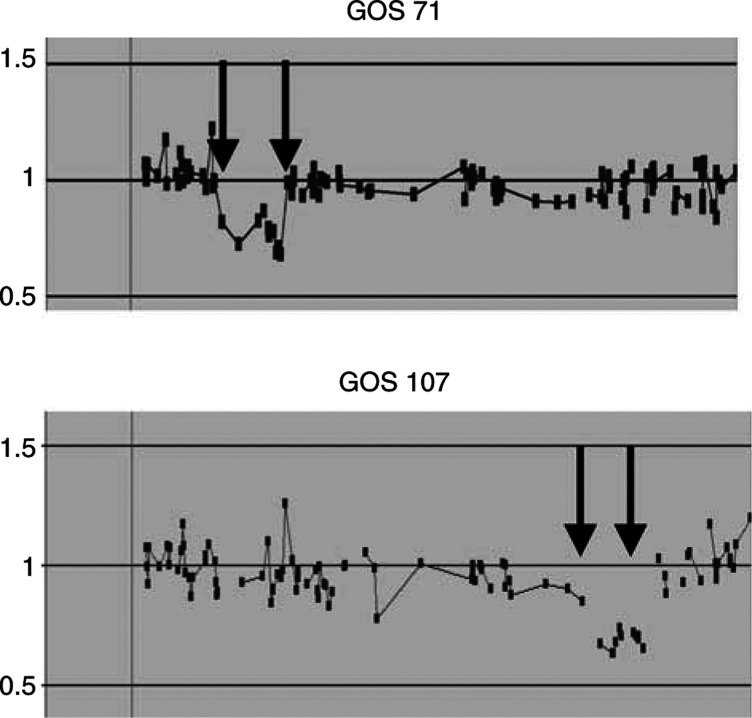
CGHa profiles for patients reportedly with 13q- syndrome. In GOS 71, the deletion (arrows) is seen in the 13q12–14 region, whereas, in patient GOS 107, the deletion (arrows) lies more distal in the 13q31–33 region.

We next extended our CGHa analysis to patients who had been reported as having 13q- syndrome which involves various partial deletions in the q22-qter region (Luo *et al*, 2000; Gutierrez *et al*, 2001; Hewson and Carter, 2002). These patients have a well-defined set of clinical phenotypes, including mental retardation, where the deletion was generally assumed to involve the terminal region of 13q, although somatic cell hybrid studies (Hawthorn and Cowell, 1995) suggested that these were, in fact, subterminal deletions. In this CGHa study, we analysed two patients, GOS 71 and GOS 107, reportedly with 13q- syndrome (Figure 3

). The syndrome in patient GOS 71 included; hypertonia, small stature, low set posteriorly rotated ears, bilateral simian creases, metatarsus varus, cryptorchidism, high arched palate and wide alveolar margins. In contrast, the features of patient GOS 107 included, developmental delay, short stature, hearing impairment, tracheo-oesophageal fistula, renal impairment and asthma. Although the prior cytogenetic analysis had suggested the same diagnosis in these cases, the clinical phenotypes were different and CGHa analysis provided the basis for this. GOS 107 showed the typical deletion involving the 13q31–33 region, but not including the telomere, confirming that, in fact, this is an interstitial deletion which was located between BACs RP11-86c3 (89.2 Mbp) and RP11-7b23 (101.15 Mbp), which spans a 12 Mbp region. By contrast, GOS 71 showed a much more proximal deletion involving the 13q12–13 region, between BACs RP11-179a7 (33.2 Mbp) and RP11-269c23 (43.87 Mbp), a distance of 10.67 Mbp. Clearly, although there is some overlap in the clinical phenotype between these two patients, the deletions are very different, which accounts for the discrepancy in their clinical phenotype. Importantly, the original cytogenetic diagnosis for GOS 71 was misinterpreted and this deletion does not include RB1, which is located at 47.9 Mbp.

**Figure 4:**
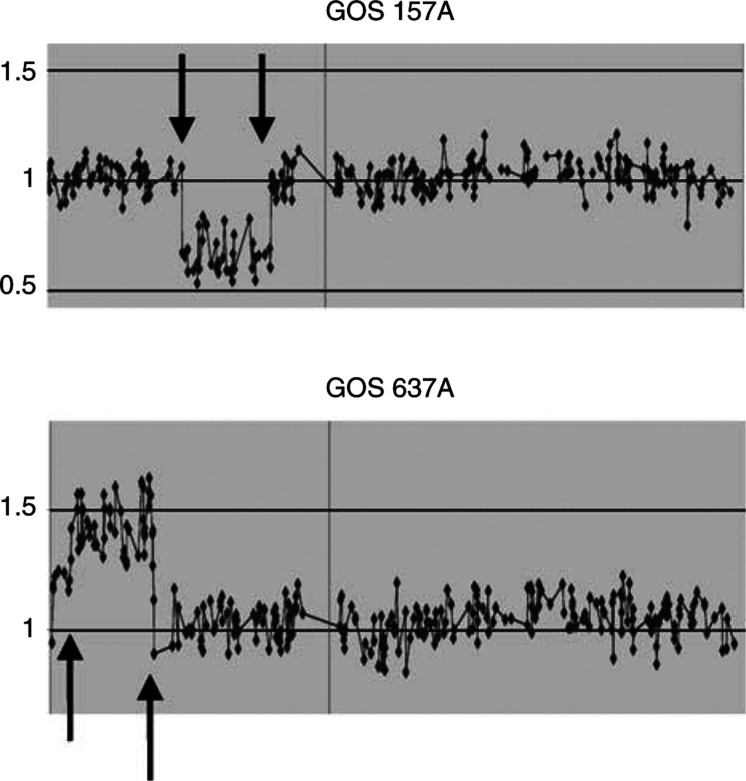
CGHa profiles for patients with constitutional chromosome abnormalities involving chromosome 11. Patient GOS 157 carries a deletion (arrows) in the 11p13 region and patient GOS 637 shows a triplication (arrows) in the 11p15 region.

The study of constitutional deletions on chromosome 13 clearly has the advantage of speed, and accuracy of the diagnosis. To extend our studies, we investigated DNA from other patients with syndrome-related chromosome abnormalities. Aniridia is a rare hereditary disease resulting in the absence of irises. In the familial form, the phenotype segregates as an autosomal dominant disorder due to mutations in the PAX6 gene (Davis and Cowell, 1993). Sporadic cases of aniridia show a 50% increased risk to the development of Wilms tumour (WT), a pediatric cancer of the kidney (Riccardi *et al*, 1978). In these patients, the cancer predisposition results from the presence of a constitutional deletion involving the 11p13 region containing both the WT1 and PAX6 genes. Patients with PAX6 gene mutations clearly represent the hereditary form of the disease, and are not at increased risk to the development of WT. From a genetic counselling standpoint, a sporadic case of aniridia could either carry a deletion predisposing to WT, or carry a *de novo* mutation in the PAX6 gene. Therefore, being able to exclude the tumour risk in these patients would involve either a mutation study of the PAX 6 gene, which is a complex and time-consuming procedure, or a cytogenetic analysis of the 11p13 region. To assess the utility of the CGHa approach in this situation, we used DNA from patient GOS 157, which we had previously demonstrated to carry a small deletion involving the 11p13 region (Cowell *et al*, 1989b). The results are shown in Figure 4

, where the presence of a heterozygous deletion is clearly seen in the short arm of chromosome 11, spanning a distance between 26.62 and 44.6 Mbp (18 Mbp), which includes the PAX6 and WT1 genes.

From the experiments described above, it is clear that CGHa can be used to quickly and efficiently identify heterozygous constitutional deletions, and could be easily extended to other syndromes such as the 15q deletion associated with Angelman/Prader Willi syndrome (Vogels and Fryns, 2002) or the 22q deletions associated with Di George syndrome (Baldini, 2002). Other genetic syndromes, however, are associated with extra copies of small chromosome regions. An example of this (Mannens *et al*, 1994) is the chromosome imbalance that is associated with the pediatric cancer predisposition syndrome, Beckwith–Wiedemann syndrome (BWS). In these cases, it has been demonstrated that duplication of a region in 11p15, which results in three copies of the 11p15.5 region, is responsible for the phenotype in some cases. BWS may sometimes be confused with the phenotypically similar Perlman's syndrome, which has a much higher frequency of cancer than BWS (Grundy *et al*, 1992), and for which no chromosome abnormality has yet been identified. To determine whether these types of chromosome aberration can also be detected using CGHa, we analysed the DNA from a patient who had been shown by extensive molecular and cytogenetic analysis to carry a nonreciprocal chromosome translocation t(5;11)(p15;p15), which resulted in the triplication of the 11p15 region (Grundy *et al*, 1998). The CGHa profile from this patient, GOS 637, is shown in Figure 4, and demonstrates that the translocation event is more probably the result of an insertion of the 11p 15.5 region spanning BACs RP11-120e20 (3.67 Mbp) and RP11-6k5 (20.37 Mbp), covering 16.77 Mbp, in the distal region of 5p15. On the log scale, all of the BACs in this region show an intensity ratio of +0.5, confirming the presence of an extra copy of this region. The most distal BACs (0–3.67 Mbp), however, show a ratio closer to 1. The most telomeric BAC on the array, RP11-123f4, is clearly only present in a diploid complement and, although the adjacent series of BACs show a ratio of 1.2, this is still within the range of ‘noise’ shown for the other BACs along the chromosome, suggesting this region is also present in only two copies. Thus, although the diagnosis of BWS is not at issue, this analysis provides valuable information about the extent, and hence the gene content, of the region involved.

Our CGHa analysis of constitutional chromosome abnormalities, therefore, provides a demonstration that heterozygous, predisposing chromosome deletions and duplications can easily be detected and accurately defined. The high resolution of this array means that small chromosome deletions, which cannot be detected using conventional chromosome analysis, will also be identified. The other advantage of CGHa is that only small amounts of DNA are required for the hybridisation, without the need for the preparation of metaphase chromosome spreads, which means that the analysis can be performed using DNA from nondividing tissue such as buccal swabs or skin biopsies. As a consequence, the diagnosis can be made in a relatively short time, since there is no need for extensive culture periods for sample preparation. One area where this advantage would be particularly useful is in the analysis of amniotic fluid cells or chronic villous samples for prenatal diagnosis of hereditary chromosome abnormality syndromes. The rapid turn around time associated with CGHa also presents clear advantages for the clinical management of these patients, and has important implications for genetic counselling.

The analysis of specific chromosome deletions has frequently led to the discovery of the gene(s) involved in the associated phenotype. The ability of CGHa to clearly define the gene content within the deleted or amplified region and to compare these observations between patients provides a rapid way of selecting candidate genes for more detailed study.

## References

[bib1] Baldini A (2002) DiGeorge syndrome: the use of model organisms to dissect complex genetics. Hum Mol Genet 11: 2363–2369 12351571 10.1093/hmg/11.20.2363

[bib2] Bruder CEG, Hirvela C, Tapia-Paez I, Fransson I, Segraves R, Hamilton G, Zhang XX, Evans DG, Wallace AJ, Baser ME, Zucman-Rossi J, Hergersberg M, Boltshauser E, Papi L, Rouleau GA, Poptodorov G, Jordanova A, Rask-Andersen H, Kluwe L, Mautner V, Sainio M, Hung G, Mathiesen T, Moller C, Pulst SM, Harder H, Heiberg A, Honda M, Niimura M, Sahlen S, Blennow E, Albertson DG, Pinkel D, Dumanski JP (2001) High resolution deletion analysis of constitutional DNA from neurofibromatosis type 2 (NF2) patients using microarray-CGH. Hum Mol Genet 10: 272–282 10.1093/hmg/10.3.271 11159946

[bib3] Cheung VG, Nowak N, Jang W, Kirsch IR, Zhao S, Chen XN, Furey TS, Kim UJ, Kuo WL, Olivier M, Conroy J, Kasprzyk A, Massa H, Yonescu R, Sait S, Thoreen C, Snijders A, Lemyre E, Bailey JA, Bruzel A, Burrill WD, Clegg SM, Collins S, Dhami P, Friedman C, Han CS, Herrick S, Lee J, Ligon AH, Lowry S, Morley M, Narasimhan S, Osoegawa K, Peng Z, Plajzer-Frick I, Quade BJ, Scott D, Sirotkin K, Thorpe AA, Gray JW, Hudson J, Pinkel D, Ried T, Rowen L, Shen-Ong GL, Strausberg SL, Birney E, Callen DF, Cheng JF, Cox DR, Doggett NA, Carter NP, Eichler EE, Haussler D, Korenberg JR, Morton CC, Albertson D, Schuler G, de Jong PJ, Trask BJ (2001) Integration of cytogenetic landmarks into the draft sequence of the human genome. Nature 409: 953–958 11237021 10.1038/35057192 PMC7845515

[bib4] Cowell JK, Barnett GH, Nowak NJ (2004b) Characterization of the 1p/19q chromosomal loss in oligodendrogliomas using CGHa. J Neuropath Exp Neurol, in press 10.1093/jnen/63.2.151 14989601

[bib6] Cowell JK, Hungerford J, Rutland P, Jay M (1987) A chromosomal breakpoint which separates the esterase-D and retinoblastoma predisposition loci in a patient with del(13) (q14-q31). Cancer Genet Cytogenet 27: 27–31 3472646 10.1016/0165-4608(87)90256-1

[bib5] Cowell JK, Hungerford J, Rutland P, Jay M (1989a) Genetic and cytogenetic analysis of patients showing reduced esterase-D levels and mental retardation from a survey of 500 individuals with retinoblastoma. Ophthal Ped Genet 110: 117–127 10.3109/13816818909088352 2779982

[bib7] Cowell JK, Jaju R, Kempski H (1994) Isolation and characterisation of a panel of cosmids which allow unequivocal identification of chromosome deletions involving the RB1 gene using fluorescence *in situ* hybridisation. J Med Genet 31: 334–337 8071962 10.1136/jmg.31.4.334 PMC1049810

[bib8] Cowell JK, Matsui S, Wang J, LaDuca J, Conroy J, McQuaid D, Nowak NJ (2004a) Application of comparative genome hybridization using BAC arrays (CGHa) and spectral karyotyping (SKY) to the analysis of glioblastoma multiforme. Cancer Genet Cytogenet, in press 10.1016/j.cancergencyto.2003.09.012 15120909

[bib9] Cowell JK, Nowak NJ (2004) High resolution analysis of genetic events in cancer cells using BAC arrays and CGHa. Adv Cancer Res, in press 10.1016/s0065-230x(03)90003-0 14710948

[bib10] Cowell JK, Rutland P, Jay M, Hungerford J (1986a) Deletions of the esterase-D locus from a survey of 200 retinoblastoma patients. Hum Genet 72: 164–167 3943870 10.1007/BF00283938

[bib11] Cowell JK, Rutland P, Jay M, Hungerford J (1986b) Effect of the esterase-D genotype on its *in vitro* enzyme activity. Hum Genet 74: 298–301 3465678 10.1007/BF00282552

[bib12] Cowell JK, Wadey RB, Buckle B, Pritchard J (1989b) The aniridia–Wilms' tumour association: molecular and genetic analysis of chromosome deletions on the short arm of chromosome 11. Hum Genet 82: 123–126 2542153 10.1007/BF00284042

[bib13] Davis A, Cowell JK (1993) Mutations in the PAX6 gene in patients with hereditary aniridia. Hum Mol Genet 2: 2093–2097 8111379 10.1093/hmg/2.12.2093

[bib14] Fiegler H, Carr P, Douglas EJ, Burford DC, Hunt S, Smith J, Betrie D, Gorman P, Tomlinson IPM, Carter NP (2003) DNA microarrays for comparative genomic hybridization based on DOP-PCR amplification of BAC and PAC clones. Genes Chromosomes Cancer 36: 361–374 12619160 10.1002/gcc.10155

[bib15] Grundy RG, Aledo R, Cowell JK (1998) Characterization of the breakpoints in unbalanced t(5;11)(p15;p15) constitutional chromosome translocations in two patients with Beckwith–Wiedemann syndrome using fluorescence *in situ* hybridization. Int J Mol Med 1: 801–808 9852299 10.3892/ijmm.1.5.801

[bib16] Grundy RG, Pritchard J, Baraitser M, Risdon A, Robards M (1992) Perlman and Wiedemann–Beckwith syndromes: two distinct conditions associated with Wilms' tumour. Eur J Pediatr 151: 895–898 1361910 10.1007/BF01954125

[bib17] Gutierrez J, Sepulveda W, Saez R, Carstens E, Sanchez J (2001) Prenatal diagnosis of 13q- syndrome in a fetus with holoprosencephaly and thumb agenesis. Ultrasound Obstet Gynecol 17: 166–168 11320988 10.1046/j.1469-0705.2001.00335.x

[bib18] Hawthorn LA, Cowell JK (1995) Integration of the physical and genetic linkage map for human chromosome 13. Genomics 27: 399–404 7558019 10.1006/geno.1995.1069

[bib19] Hewson MP, Carter JM (2002) Severe congenital factor VII deficiency associated with the 13q deletion syndrome. Am J Hematol 71: 232–233 12410585 10.1002/ajh.10237

[bib20] Hodgson G, Hager JH, Volik S, Hariono S, Wernick M, Moore D, Nowak N, Albertson DG, Pinkel D, Collins C, Hanahan D, Gray JW (2001) Genome scanning with array CGH delineates regional alterations in murine islet carcinomas. Nat Genet 29: 459–464 11694878 10.1038/ng771

[bib21] Horai S, Matsunaga E (1984) Differential enzyme activities in human esterase D phenotypes. Hum Genet 66: 168–170 6714977 10.1007/BF00286594

[bib22] Kempski HM, Cowell JK (1993) Detection of submicroscopic chromosomal deletions in aniridia patients using fluorescence *in situ* hybridisation and a panel of cosmids covering the WT1 gene. Int J Oncol 3: 937–940 21573456 10.3892/ijo.3.5.937

[bib23] Luo J, Balkin N, Stewart JF, Sarwark JF, Charrow J, Nye JS (2000) Neural tube defects and the 13q deletion syndrome: evidence for a critical region in 13q33–34. Am J Med Genet 91: 227–230 10756348 10.1002/(sici)1096-8628(20000320)91:3<227::aid-ajmg14>3.0.co;2-i

[bib24] Mannens M, Hoovers JMN, Redeker E, Vergjaal M, Feinberg AP, Little P, Boavida M, Coade N, Steenman M, Bliek J, Niikawa N, Tononki H, Nakamura Y, de Boer EG, Slater RM, John R, Cowell JK, Junien C, Henry I, Tommerup N, Weksberg R, Pueschel SM, Leschot NJ, Westerveld A (1994) Parental imprinting of human chromosome region 11p15.3-pter involved in the Beckwith–Wiedemann syndrome and various human neuoplasia. Eur J Hum Genet 41: 1–22 10.1159/000472337 7913866

[bib25] Mitchell CD, Cowell JK (1988) Molecular evidence that the esterase-D gene lies proximal to the retinoblastoma susceptibility locus in chromosome region 13q14. Hum Genet 81: 57–60 3198126 10.1007/BF00283730

[bib26] Riccardi VM, Sujansky E, Smith AC, Francke U (1978) Chromosomal imbalance in the Aniridia–Wilms' tumor association: 11p interstitial deletion. Pediatrics 61: 604–610 208044

[bib27] Snidjers A, Nowak N, Seagraves R, Blackwood S, Brown N, Conroy J, Hamilton G, Hindle A, Huey B, Kimura K, Law S, Myambo K, Palmer J, Yistra B, Yue J, Gray J, Jain A, Pinkel D, Albertson D (2001) Assembly of microarrays for genome-wide measurement of DNA copy number by CGH. Nat Genet 29: 263–264 11687795 10.1038/ng754

[bib28] van Heyningen V, Bobrow M, Bodmer WF, Povey S, Gardiner SE, Hopkinson DA (1975) Assignment of the genes for human mitochondrial malate dehydrogenase to chromosome 7, for mannose phosphate isomerase and pyruvate kinase to chromosome 15, and, probably, for human esterase-D to chromosome 13 using man–mouse hybrids. Cytogenet Cell Genet 14: 353–357 1192815 10.1159/000130381

[bib29] Veltman JA, Fridlyand J, Pejavar S, Olshen AB, Korkola JE, DeVries S, Carroll P, Kuo WL, Pinkel D, Albertson D, Cordon-Cardo C, Jain AN, Waldman FM (2003a) Array-based comparative genomic hybridization for genome-wide screening of DNA copy number in bladder tumors. Cancer Res 63: 2872–2880 12782593

[bib30] Veltman JA, Jonkers Y, Nuijten I, Janssen I, van der Vliet W, Huys E, Vermeesch J, Van Buggenhout G, Fryns J-P, Admiraal R, Terhal P, Lacombe D, van Kessel AG, Smeets D, Schoenmakers EFPM, van Tavenswaaij-Arts CM (2003b) Definition of a critical region on chromosome 18 for congenital aural atresia by ArrayCGH. Am J Hum Genet 72: 1578–1584 12740760 10.1086/375695 PMC1180319

[bib31] Veltman JA, Schoenmakers EFPM, Eussen BH, Janssen I, Merkx G, van Cleef B, van Ravenswaaij CM, Brunner HG, Smeets D, van Kessel AG (2002) High-throughput analysis of subtelomeric chromosome rearrangements by use of array-based comparative genomic hybridization. Am J Hum Genet 70: 1269–1276 11951177 10.1086/340426 PMC447601

[bib32] Vogels A, Fryns JP (2002) The Prader–Willi syndrome and the Angelman syndrome. Genet Couns 13: 385–396 12558108

[bib33] Wessendorf S, Schwaenen C, Kohlhammer H, Kienle D, Wrobel G, Barth FEB, Nessling M, Moller P, Dohner H, Lichter P, Bentz M (2003) Hidden gene amplifications in aggressive B-cell non-Hodgkin lymphomas detected by microarray-based comparative genomic hybridization. Oncogene 22: 1425–1429 12618769 10.1038/sj.onc.1206297

[bib34] Wilhelm M, Veltman JA, Olshen AB, Jain AN, Moore DN, Presti Jr JC, Kovacs G, Waldman FM (2002) Array-based comparative genomic hybridization for the differential diagnosis of renal cell cancer. Cancer Res 62: 957–960 11861363

[bib35] Young LJ, Lee EY, To HA, Bookstein R, Shew JY, Donoso LA, Sery T, Giblin M, Shields JA, Lee WH (1988) Human esterase D gene: complete cDNA sequence, genomic structure, and application in the genetic diagnosis of human retinoblastoma. Hum Genet 79: 137–141 3164702 10.1007/BF00280552

